# Coexistence of Congenital Adrenal Hyperplasia and Autoimmune Addison's Disease

**DOI:** 10.3389/fendo.2019.00648

**Published:** 2019-09-27

**Authors:** Sigrid Aslaksen, Paal Methlie, Magnus D. Vigeland, Dag E. Jøssang, Anette B. Wolff, Ying Sheng, Bergithe E. Oftedal, Beate Skinningsrud, Dag E. Undlien, Kaja K. Selmer, Eystein S. Husebye, Eirik Bratland

**Affiliations:** ^1^Department of Clinical Science, University of Bergen, Bergen, Norway; ^2^K.G. Jebsen Center for Autoimmune Diseases, University of Bergen, Bergen, Norway; ^3^Department of Medicine, Haukeland University Hospital, Bergen, Norway; ^4^Institute of Clinical Medicine, University of Oslo, Oslo, Norway; ^5^Department of Medical Genetics, Oslo University Hospital, Oslo, Norway; ^6^Department of Radiology, Haukeland University Hospital, Bergen, Norway; ^7^Division of Clinical Neuroscience, Department of Research and Development, Oslo University Hospital, University of Oslo, Oslo, Norway; ^8^National Centre for Epilepsy, Oslo University Hospital, Oslo, Norway

**Keywords:** adrenal insufficiency, congenital adrenal hyperplasia, 3β-hydroxysteroid dehydrogenase type 2 deficiency, autoimmune adrenalitis, autoimmune Addison's disease

## Abstract

**Background:** Underlying causes of adrenal insufficiency include congenital adrenal hyperplasia (CAH) and autoimmune adrenocortical destruction leading to autoimmune Addison's disease (AAD). Here, we report a patient with a homozygous stop-gain mutation in 3β-hydroxysteroid dehydrogenase type 2 (3βHSD2), in addition to impaired steroidogenesis due to AAD.

**Case Report:** Whole exome sequencing revealed an extremely rare homozygous nonsense mutation in exon 2 of the *HSD3B2* gene, leading to a premature stop codon (NM_000198.3: c.15C>A, p.Cys5Ter) in a patient with AAD and premature ovarian insufficiency. Scrutiny of old medical records revealed that the patient was initially diagnosed with CAH with hyperandrogenism and severe salt-wasting shortly after birth. However, the current steroid profile show complete adrenal insufficiency including low production of pregnenolone, dehydroepiandrosterone (DHEA) and DHEA sulfate (DHEA-S), without signs of overtreatment with steroids.

**Conclusion:** To the best of our knowledge, this is the first description of autoimmune adrenalitis in a patient with 3βHSD2 deficiency and suggests a possible association between AAD and inborn errors of the steroidogenesis.

## Introduction

**Figure 1:**
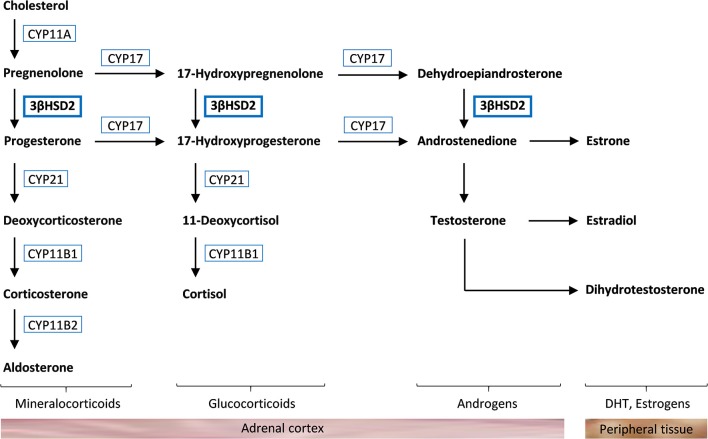
Steroidogenesis in the adrenal cortex. Cholesterol is converted to aldosterone, cortisol, and androgens through different pathways that require specific enzymes [cholesterol side-chain cleavage enzyme (CYP11A), 17α-*hydroxylase* (CYP17), 3β-hydroxysteroid dehydrogenase type 2 (3βHSD2), 21-hydroxylase (CYP21), 11β-hydroxylase (CYP11B1), and aldosterone synthase (CYP11B2)]. Androstenedione and testosterone are further converted to dihydrotestosterone (DHT) and estrogens in peripheral tissue.

3β-hydroxysteroid dehydrogenase type 2 (3βHSD2) catalyzes the conversion of Δ5-steroids into Δ4-steroids (Figure 1). Deficiency of 3βHSD2 causes a rare autosomal recessive form of congenital adrenal hyperplasia (CAH) characterized by high levels of pregnenolone, 17-hydroxypregnenolone, dehydroepiandrosterone (DHEA), DHEA sulfate (DHEA-S), and androstenediol, and lack of cortisol, aldosterone, and androstenedione (1). There are two isozymes of 3βHSD encoded by *HSD3B1* and *HSD3B2*. 3βHSD2 is expressed in the gonads and the adrenal cortex, whereas 3βHSD1 is expressed in peripheral tissues, converting circulating DHEA to testosterone (1). 3βHSD2 deficiency can therefore cause relatively high levels of testosterone in females, whereas it cannot compensate for the absence of adrenal and gonadal synthesis of testosterone in males. This causes ambiguous genitalia in males, whereas female newborns exhibit mild virilization or normal sexual differentiation, and may remain undiagnosed until a salt-wasting crisis occurs (1–3).

Adrenal insufficiency can also be due to autoimmune adrenalitis, or autoimmune Addison's disease (AAD), characterized by an immunological attack of the adrenal cortex leading to decreased production of cortisol and aldosterone (4). The self-antigen 21-hydroxylase (21OH) is the dominant target of adrenal autoantibodies and autoreactive T cells (4). Therefore, autoantibodies against 21OH and low serum cortisol levels are important diagnostic markers for AAD.

To the best of our knowledge, we here report the first patient affected by both inborn 3βHSD2 deficiency and acquired AAD.

## Case Report

**Figure 2:**
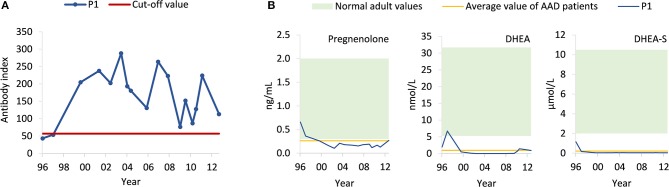
Levels of circulating 21OH autoantibodies in the patient (P1) and steroid profiling. **(A)** Using radioimmunoprecipitation assay, levels of 21OH autoantibodies (blue) were measured in serum samples taken at different time points from 1996 to 2013. The cut-off value (red) was set to obtain the maximal accuracy as calculated by an interlaboratory study (6). **(B)** Using ELISA, levels of pregnenolone, DHEA, and DHEA-S were measured in serum samples from the patient (P1, blue) taken from different time points from 1996 to 2013. Normal adult values (green) are based on established reference values from the textbook “Gynecologic Endocrinology” (pregnenolone) (7), hormone laboratory at Oslo University Hospital (DHEA) (https://ehandboken.ous-hf.no/api/File/GetFile?entityId=105475) and hormone laboratory at Haukeland University Hospital (DHEA-S) (https://analyseoversikten.no/analyse/14). The average value of AAD patients (yellow) is measured from serum samples of patients included in our biobank.

A whole-exome sequencing study involving 142 AAD patients (5) revealed a patient with a rare homozygous mutation in exon 2 of *HSD3B2* [frequency ~0.00003 in the Genome Aggregation Database (gnomAD)] at nucleotide position 15 (NM_000198.3:c.15C>A), resulting in the exchange of the cysteine codon to a premature stop codon (p.Cys5Ter). Subsequent screening of the Norwegian Addison Registry identified the patient harboring the mutation, a 55-year-old female with AAD, premature ovarian insufficiency and vitamin B12 deficiency, accompanied by 21OH- and parietal cell autoantibodies (Figure 2A). Notably, she did not carry any of the major histocompatibility complex (MHC) alleles conferring high risk to develop AAD (4). She tested negative for other autoantibodies such as anti-thyroid peroxidase, and had normal levels of thyroid stimulating hormone (0.40–4.50 mIU/L). Scrutiny of early medical records showed that she exhibited hyperpigmentation of genitalia and clitoris hypertrophy already at birth. One week of age, she started to vomit and developed hyponatremia (127 mmol/L) and hyperkalemia (6.1 mmol/L). Elevated levels of 17-ketosteroids were detected. She was therefore diagnosed with CAH and supplemented with cortisone acetate and eventually fludrocortisone. Her sister had also been diagnosed with CAH, but unfortunately died in an adrenal crisis at 2 years of age.

Given these conflicting findings, computer tomography (CT) and magnetic resonance (MR) scans, taken from age 43 to 51, were re-evaluated and the adrenals were found to be in the lower range of normal thickness 2–3 mm, indicating adrenocortical atrophy rather than hyperplasia as would be expected in case of isolated CAH. We then obtained a steroid profile by liquid chromatography tandem mass spectroscopy (LC-MS/MS) of a serum sample taken after an overnight medication fast (Table 1). Levels of all mineralocorticoids, and most glucocorticoids and androgens were below the detection limit, except for tetrahydrocortisol (0.308 nmol/L), 5α-tetrahydrocortisol (0.187 nmol/L), and testosterone (0.066 nmol/L). Total pregnenolone, DHEA and DHEA-S levels were measured by enzyme-linked immunosorbent assay (ELISA) from previously collected serum samples spanning the years 1996–2013 (Figure 2B). Normal levels of pregnenolone and DHEA were found in samples from the first 2 years, but then levels decreased toward the subnormal levels generally seen in AAD patients. DHEA-S was also below the normal range, typical of AAD patients, at all time points. ACTH was measured at several time points, revealing elevated levels on multiple occasions. The highest levels were observed in 2011 at 130 pmol/L (normal range 2.0–11.6 pmol/L). Importantly, we detected no subsequent increase in pregnenolone, DHEA, and DHEA-S in spite of elevated ACTH levels (Figure 2B).

**Table 1:** Steroid profile of the patient.

**Steroids**	**LLoQ (nM)**	**Patient serum sample**
Progesterone	0.114	<LLoQ
11-deoxycorticosterone	0.023	<LLoQ
Corticosterone	0.114	<LLoQ
Tetrahydrocorticosterone	0.114	<LLoQ
18-hydroxycorticosterone	0.069	<LLoQ
Aldosterone	0.0023	<LLoQ
Tetrahydroaldosterone	0.062	<LLoQ
17-hydroksyprogesterone	0.114	<LLoQ
11-deoxycortisol	0.114	<LLoQ
21-deoxycortisol	0.023	<LLoQ
Cortisol	0.914	<LLoQ
Tetrahydrocortisol	0.114	0.308
5α-tetrahydrocortisol	0.114	0.187
18-hydroxycortisol	0.046	<LLoQ
18-oxocortisol	0.046	<LLoQ
Cortisone	0.914	<LLoQ
Tetrahydrocortisone	0.343	<LLoQ
5α-tetrahydrocortisone	0.343	<LLoQ
Dehydroepiandrosterone	0.617	<LLoQ
Dehydroepiandrosterone sulfate	22.862	<LLoQ
Androstenedione	0.023	<LLoQ
Testosterone	0.023	0.066
Dihydrotestosterone	0.023	<LLoQ
Epitestosterone	0.023	<LLoQ

*Overview of the levels of 24 analytes in a fasting serum sample from the patient, including the Lower Limit of Quantification Levels (LLoQ) for each analyte. The analysis was performed using LC-MS/MS*.

She is currently, at age 57, treated with 20 mg hydrocortisone (Plenadren™) and 100 μg fludrocortisone, and does not have suppressed ACTH-values.

## Discussion

This is the first report of a patient with primary adrenal insufficiency due to both 3βHSD2 deficiency and AAD. At inclusion in the national Addison registry, she was classified as having AAD with vitamin B12 deficiency, and 21OH- and parietal cell autoantibodies. However, genetic screening and scrutiny of old records revealed a rare form of CAH due to a stop-gain mutation in *HSD3B2*. The presence of small sized adrenal glands instead of hyperplastic glands, normally seen in CAH, and no overproduction of *Δ*5-steroids following elevated ACTH levels, suggest that the adrenal cortex is not functioning. Presence of tetrahydrocortisol and 5α-tetrahydrocortisol is consistent with her ongoing replacement therapy which includes hydrocortisone. The low, but detectable, level of testosterone may be due to conversion of DHEA by 3βHSD1 in the periphery. Previously measureable pregnenolone levels suggest that the autoimmune destruction of the adrenal cortex commenced sometimes in the 1990-ties.

According to literature, we could only find one reported case of CAH occurring together with complete adrenal cortex insufficiency suspected to be autoimmune adrenalitis. This patient, however, had neither 21OH autoantibodies, nor MHC risk alleles, but was positive for autoantibodies against 17α-hydroxylase (8). Therefore, we speculate there might be other rare unreported cases of autoimmune adrenalitis due to early diagnosis of CAH, masking the clinical symptoms of AAD. Interestingly, several other AAD patients included in our exome sequencing analysis carry rare heterozygous non-synonymous variants in *HSD3B2* (Table 2). Although it appears that family members of CAH patients, carrying heterozygous *HSD3B2* mutations, maintain normal 3βHSD2 activity *in vivo* (14), genetic variations in *HSD3B2* are associated with other conditions such as idiopathic hypospadias and prostate cancer (15, 16). Therefore, both subtle molecular abnormalities and deleterious mutations in *HSD3B2* could have biological consequences, and may play a role in the pathogenesis of the immune-mediated adrenocortical destruction in AAD.

**Table 2:** Overview of rare variants of *HSD3B2* (NM_000198.3) found in AAD patients.

**Mutation annotation**				**Frequencies**				***In silico*** **prediction tools**				
***HSD3B2* variant**	**Protein change**	**Chromosomal location** **NC_000001.10** **GRCh37 (hg19)**	**Exon number**	**AAD cohort**	**In-house exome database**	**NCGC^a^**	**gnomAD^b^**	**SIFT^c^**	**PolyPhen2^c^**	**Mutation taster^c^**	**PROVEAN^c^**	**CADD^c^**
c.15C>A	p.Cys5Ter	g.119958057C>A	2	0.0076	0	0	0.000032^d^	NA	NA	Disease causing	NA	36.0
c.707T>C	p.Leu236Ser	g.119964831T>C	4	0.0038	0	0	0.0038	Tolerated	Benign	Polymorphism	Neutral	13.74
c.931C>T	p.Gln311Ter	g.119965055C>T	4	0.0038	0	0	0.0000040	NA	NA	Disease causing	NA	34.0
c.995A>C	p.Lys332Thr	g.119965119A>C	4	0.0038	0	0	0	Deleterious	Probably damaging	Disease causing	Deleterious	25.0

a *NCGC, Norwegian Cancer Genomics Consortium exome database, http://invitro.hpc.uio.no:8082/vcf-miner/*.

b *gnomAD, The Genome Aggregation Database, https://gnomad.broadinstitute.org/*.

c *In silico variant pathogenicity predictors: SIFT (9), PolyPhen2 (10), MutationTaster2 (11), PROVEAN (12), and CADD (Combined Annotation Dependent Depletion) scores above 20 indicate that a variant is amongst the top 1% of deleterious variants in the human genome (13)*.

d *The variant has previously been reported in gnomAD, but never in a homozygous state as in this case report*.

## Data Availability Statement

This manuscript contains previously unpublished data. The name of the repository and accession number(s) are not available.

## Ethics Statement

The studies involving human participants were reviewed and approved by Regional Committee for Medical and Health Ethics. The patients/participants provided their written informed consent to participate in this study. Written informed consent was obtained from the individual(s) for the publication of any potentially identifiable images or data included in this article.

## Author Contributions

All authors listed have made a substantial, direct and intellectual contribution to the work, and approved it for publication.

### Conflict of Interest

The authors declare that the research was conducted in the absence of any commercial or financial relationships that could be construed as a potential conflict of interest.
